# Evaluation of static and dynamic balance in athletes with anterior cruciate ligament injury – A controlled study

**DOI:** 10.6061/clinics/2016(08)03

**Published:** 2016-08

**Authors:** Tiago Lazzaretti Fernandes, Ellen Cristina Rodrigues Felix, Felipe Bessa, Natália MS Luna, Dai Sugimoto, Júlia Maria D’Andrea Greve, Arnaldo José Hernandez

**Affiliations:** I Faculdade de Medicina da Universidade de São Paulo, Instituto de Ortopedia e Traumatologia, Grupo de Medicina do Esporte - FIFA, São Paulo/SP, Brazil; II Faculdade de Medicina da Universidade de São Paulo, Instituto de Ortopedia e Traumatologia, Laboratório de Cinesiologia (LEM), São Paulo/SP, Brazil; III Harvard Medical School, Massachusetts General Hospital, Department of Orthopedic Surgery, Bioengineering Laboratory, Boston, MA, USA; IV Boston Children’s Hospital, Department of Orthopedics, Division of Sports Medicine, Boston, MA, USA; V The Micheli Center for Sports Injury Prevention, Waltham, MA, USA

**Keywords:** Anterior Cruciate Ligament, Postural Balance, Knee

## Abstract

**OBJECTIVES:**

Anterior cruciate ligament injury leads to adaptive responses to maintain postural control. However, there is no consensus regarding whether leg dominance also affects postural control in athletes with anterior cruciate ligament injury. The purpose of this study was to evaluate dynamic and static postural control among athletes with and without anterior cruciate ligament injury to the dominant leg.

**METHODS:**

Twenty-eight athletes, twenty-one males and seven females aged 15-45 years, were allocated to one of two groups: the anterior cruciate ligament injury group (26±3 years) or the control group without anterior cruciate ligament injury (25±6.5 years). All subjects performed one legged stance tests under eyes open and eyes closed conditions and squat and kick movement tests using a postural control protocol (AccuSway*^Plus^* force platform, Massachusetts). The center of pressure displacement and speed were measured by the force platform. In addition, the distance traveled on the single-leg hop test was assessed as an objective measure of function.

**RESULTS:**

Significantly greater mediolateral sway was found under the eyes closed condition (*p*=0.04) and during squat movement (*p*=0.01) in the anterior cruciate ligament injury group than in the control group. Analysis of the single-leg hop test results showed no difference between the groups (*p*=0.73).

**CONCLUSION:**

Athletes with anterior cruciate ligament injury had greater mediolateral displacement of the center of pressure toward the dominant leg under the eyes closed condition and during squat movement compared to control athletes.

## INTRODUCTION

Anterior cruciate ligament (ACL) injury is very disabling for various sporting activities 1 because it causes knee instability with functional deficits and damages other joint structures such as the meniscus. Leg dominance has been described as an important factor in balance after an ACL injury not only because of the adaptive changes that occur, but also because of limb asymmetries in muscle strength, muscle activation and balance 2–4.

These functional impairments lead to the need for surgical treatment, which involves absence from work and physical activities for extended periods 1,5. Studies show that even after surgical intervention for an ACL injury, proprioceptive deficits remain 6. This loss of sensory information causes a postural control deficit and functional asymmetry 7.

Postural control can be evaluated on a force platform using static and dynamic measurements and single-limb tests are an ideal assessment method for this purpose in these patients 8–10. The force platform is an optimal tool for quantitative assessment of postural balance. It provides information about spatial and temporal alterations in body position for maintenance of balance in the vertical and horizontal axes and these data are used to calculate oscillations in the center of pressure (COP) in the anteroposterior and mediolateral directions, as well as the velocity of the oscillations and the sway area (COP) 10,9,11,12. Several studies compared static balance under the eyes open condition between individuals with and without ACL injury 2,13 and showed no differences. Other studies evaluating the COP have examined movements such as squatting and kicking on a force platform 2,14.

Functional impairments are evaluated using a series of hop test 15,16. This test is used to assess dynamic postural control, as there is a relation between the hop test score and COP displacement during a task. This test has been widely used in studies of patients with ACL injury who have undergone reconstructive surgery and has been employed by physicians and healthcare providers to support decision making regarding the ability to return to sporting activities because this test serves as a reliable and valid performance-based outcome measure for patients 17–19.

It is important to investigate whether there are differences in balance and functional test results between groups with and without ACL injury, as such differences may impact clinical decision making regarding the ability to return to sporting activities 7. Additionally, the ACL injury group may exhibit reduced performance on both tests, as ACL injury causes knee instability with functional deficits and damages other joint structures, such as the meniscus.

Therefore, the objective of this study was to evaluate stabilometric and functional parameters in athletes with and without ACL injury to the dominant leg.

## MATERIALS AND METHODS

A total of 28 athletes, 14 with ACL injury to the dominant leg and 14 without ACL injury, were included in this transversal study. This study was performed at the Laboratório de Estudo do Movimento do Instituto de Ortopedia e Traumatologia, Hospital das Clínicas da Faculdade de Medicina da Universidade de São Paulo (HCFMUSP), between August 2012 and May 2013. This study was approved by the Ethics Committee of the Instituto de Ortopedia e Traumatologia of the Faculdade de Medicina da Universidade de São Paulo (FMUSP) and all patients signed an informed consent form prior to this experiment.

The athletes were allocated to one of the following two groups: control group (athletes without ACL injury - 10 men and 4 women) or ACL injury group (11 men and 3 women). For comparisons and baseline assessments, the following demographic data were collected: age, sex, body mass and height (Table 1).

The inclusion criteria were recreational or professional athletes aged between 15 and 45 years, sports training for at least twelve months, Tegner activity level of five or above and no history of a medical problem that limited activities within 6 weeks prior to the experiment. Additionally, primary injury to the ACL was an inclusion criterion for the ACL injury group.

The exclusion criteria were history of previous knee injury or leg surgery, associated lesion in the meniscus or cartilage, other ligament injury and valgus or varus knee alignment. Additionally, knee instability was an exclusion criterion for the control group.

The ACL injury group consisted a consecutive series of injured athletes who did not undergo ACL reconstruction surgery and the time from ACL injury to this experiment was greater than three months to avoid the inflammatory period, during which functional disability is greatest, range of motion is restricted and swelling is present. Orthopedists at the study center assessed the volunteers via magnetic resonance imaging, clinical maneuvers and history of recurrence of knee instability. The control group was selected to match the characteristics of the ACL injury group features. All the athletes followed the same rehabilitation protocol.

Athletes were evaluated on an AccuSway^Plus^ force platform (AMTI, Massachusetts, USA).

**Figure 1:**
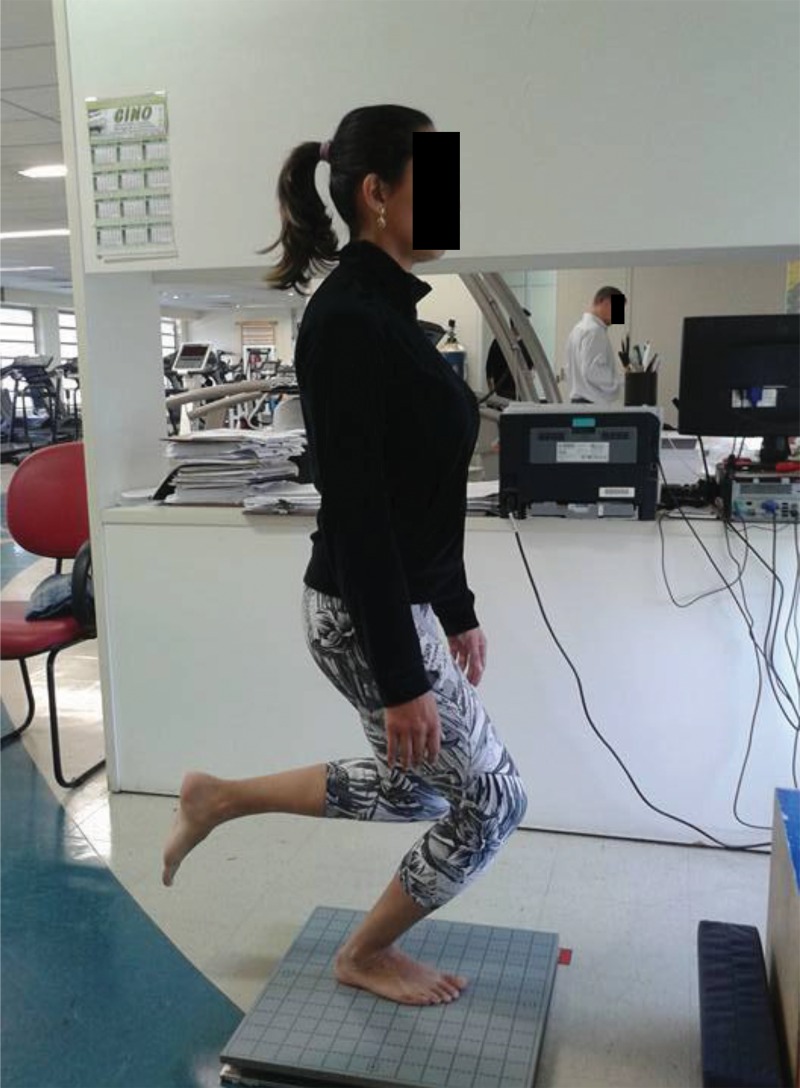
Flexion/extension test (squat movement).

**Figure 2:**
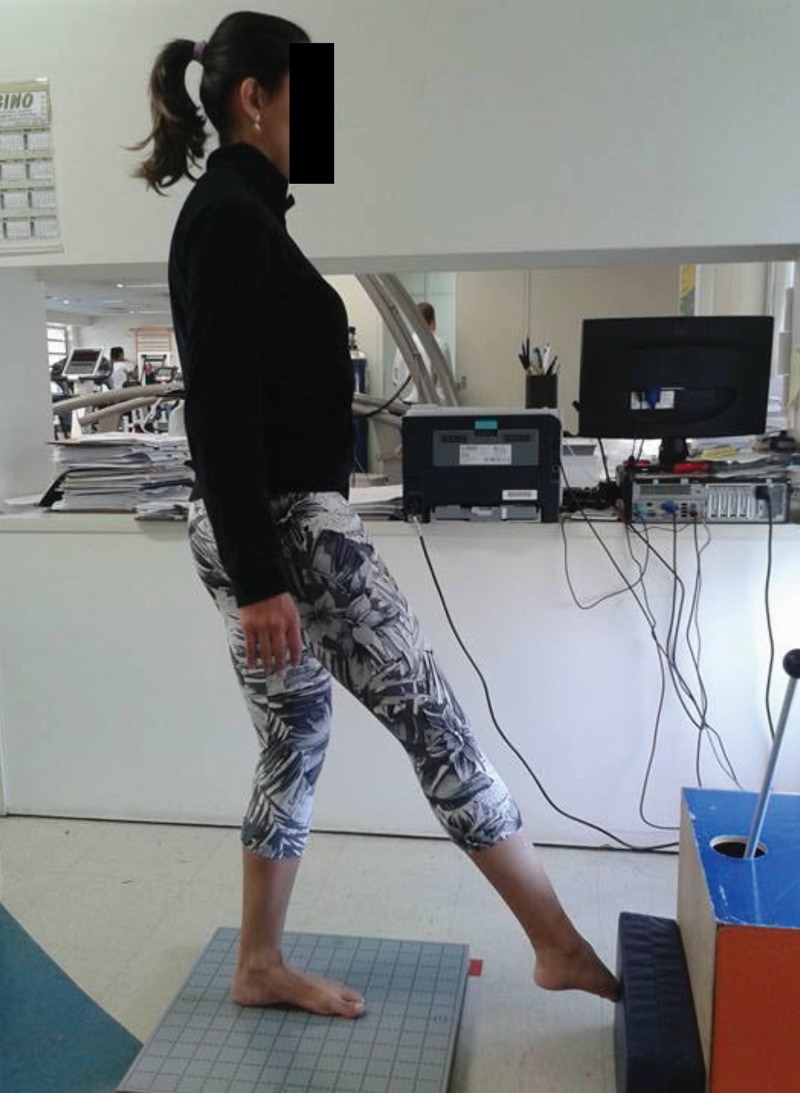
Kick test (rotational movement).

Four tests were performed, all with single-leg support: eyes open, eyes closed, knee flexion up to 45^o^ (a squat associated with a straight trunk position) (Figure 1) and rotation of the trunk and hips (simulating a kick with the outside edge of the foot) (Figure 2). The last test was performed through internal rotation of the trunk on the supporting limb and the movement was continuously repeated during the test period. The movement speed was established according to the strategy adopted by each volunteer.

Each test was conducted three times. The tests on single-leg support with eyes open and eyes closed lasted 30 seconds. The knee flexion movement (squat) and kick simulation tests lasted 10 seconds. There was an interval of 30 seconds between each test. The arithmetic average result of the three tests was calculated.

The following variables were obtained: maximum displacement of the center of pressure (COP in the anteroposterior direction (cm); maximum displacement of the COP in the mediolateral direction (cm); velocity of oscillation (cm/second); and area of displacement (95% of the area formed by the ellipse of the trajectory from the COP).

Patients also performed the single-leg hop distance test, which consists of a horizontal jump for the farthest possible distance using only one leg while keeping the hands behind the body. Three jumps were performed with each leg, with an interval of 1 minute between jump, and the arithmetic average distance of each jump was calculated.

### Statistical Analysis

Demographic data were calculated using Student’s T-test and Fisher’s exact test.

For hop-test analysis and static and dynamic evaluations using the force platform, the Shapiro-Wilk normality test was performed. Student’s T-tests were used for parametric values and the Mann-Whitney test was used for non-parametric values. All the tests were performed considering an alpha level of 0.05 and a power of 80% (SPSS-9 for Windows).

### RESULTS

There were no statistically differences in the baseline results between the two groups; the means and standard deviations are displayed in Table 1. The Tegner activity level was 7 in both groups and the time after injury ranged from 3 to 18 months (mean=9.29±9.59 months).

There was no difference in horizontal hop test performance between the two groups (ACL injury group=114.8±40.3 cm and control group=109.8±35.1 cm; *p*=0.73, Mann-Whitney test).

In the eyes closed test, 6 athletes in the ACL injury group and 9 in the control group did not complete the test (they did not maintain their balance for 30 seconds all 3 times).

There was a difference in the eyes closed test results and in the knee flexion test (squat) results between the control and ACL injury groups (Mann-Whitney test), as shown in Table 2.

The ACL injury group exhibited greater mediolateral displacement than the control group based on the single-leg test with eyes closed and the dynamic knee flexion test. The effect sizes (Table 2) were small to large in eyes open condition, negligible in the eyes closed condition and small to large in the tests of dynamics: flexion/extension (squat) and kick.

## DISCUSSION

The main finding of this study is that static postural control is decreased during the one-legged stance test with eyes closed and during squat movement requiring dynamic balance on only the dominant leg in the ACL injury group compared to the control group. Under static conditions, the athletes with ACL injury exhibited greater mediolateral displacement than the control athletes.

As the visual and vestibular systems contribute to the correction and maintenance of static and dynamic postural balance 24,21, it is possible that the loss of proprioception related to ACL injury was exacerbated by a lack of visual afferents. This could explain the poorer test performance of the subjects under eyes closed conditions than under eyes open conditions 1,20.

Few studies have investigated postural control following ACL injury. Using a similar method, Lysholm et al. 21 observed a change in anteroposterior position of the COP under eyes open conditions in chronic ACL injury patients. Additionally, Tecco et al. 20 reported greater anterior and medial displacements under dynamic eyes-closed conditions in patients with ACL injury than in non-uninjured athletes. These studies used different evaluation conditions: in the former, body sway was measured with perturbation several years after injury and in the latter, the evaluation was performed under two different dental occlusal conditions. Alternatively, in this study, the time after injury was between 3 and 18 months and the tests were performed without perturbation.

As ACL injury enables greater translation of the anterior tibia, an increase in displacement of the COP in the anteroposterior direction would be expected after ACL injury. For anatomical reasons, the knee has a limited capacity to make postural adjustments in the frontal plane 21. Tecco et al. 20 suggested that dental occlusion could improve postural control due to participation of the stomatognatic system in maintaining balance.

Nevertheless, based on the results of this study, a powerful action of the soleus and quadriceps muscles during knee flexion should prevent greater oscillation in this direction 22.

Mediolateral displacement (the path of the COP in the coronal plane) is related to the maintenance of pelvic balance (lateral tilt) during single-leg support and may be more dependent on visual control than anteroposterior displacement, which could explain the greater displacement on the static test under the eyes closed condition than under the eyes open condition. In the knee flexion test (squat), the observation of the greatest displacement may be associated with the inability to conduct compensatory postural adjustments by coordinating muscle responses to rotate the hips on the anteroposterior axis due to the body position during the task. Consequently, there is greater increased displacement in the coronal plane to maintain the center of gravity within a small base. Eccentric action of the quadriceps might introduce better control of anteroposterior displacement because the knee flexion movement was performed slowly, preventing greater control in the sagittal plane 23,24.

Several studies reported the use of dynamic tests such as kick and squat tests. Santos et al. 14 investigated anticipatory and compensatory postural adjustment, which is associated with postural control in individuals with chronic ankle instability, using a kick test on a force platform. The subjects were instructed to kick a ball, but the movement was not a kick with hip rotation as performed in the present study. The authors intended to replicate perturbation training commonly used at athletic clubs and during rehabilitation. They found greater mediolateral range and velocity of COP excursion in individuals with chronically unstable ankles than in those with healthy ankles. Their result showed that the task was sufficiently challenging to reveal a difference between groups with and without ankle instability. Kim et al. 25 investigated how gender and age affect postural control during the dynamic squat test and demonstrated that COP sway increases with age only among females, but COP sway was similar between genders among young individuals; only uninjured subjects were investigated by these authors. They proposed that musculoskeletal factors are the cause of this gender difference.

In the present study, we did not find a significant difference in hop test performance between the groups. The single-leg hop test is one of the most commonly used tests to evaluate knee stability after ACL injury 16,17,26,27. Previous studies in the literature reported that patients with ACL injury performed worse on this test and that reconstruction surgery recovered the function of ACL injury patients. We believe that our population with ACL deficiency was composed by athletes with a greater interval since ACL injury than other studies.

One limitation of this study is the difficulty in comparing the results of this study with those of other studies using the same methodology of measuring dynamic performance on a force platform as performed in the present study. Another limitation is that muscle strength, degree of instability (KT -1000), knee valgus and kinematics parameters on the tests were not evaluated. We suggest that future studies should include the performance of dynamic evaluation as performed in this study, as well as the use of functional tests and kinematics measures. This information would improve the quality of the data for parameters related to COP displacement and function of individuals with ACL injury.

Concluding, athletes with ACL injury had greater lateral displacement of the COP under the closed eyes condition and during squat movement on the dominant leg than control athletes.

## AUTHOR CONTRIBUTIONS

Fernandes TL, Hernandez AJ, Greve JM and Luna NM contributed to the conception and design of the study, analysis and interpretation of the data, drafting and revision of the manuscript. Felix ECR was responsible for acquisition, analysis and interpretation of the data and drafting and revision of the manuscript. Bessa F and Sugimoto D contributed to drafting and revision of the manuscript. We declare that all the listed authors actively participated in this study and approved the final version of the manuscript.

## Figures and Tables

**Table 1:** Demographic data of the control and anterior cruciate ligament injury groups.

	Control (N=14, male=10, female=4)	ACL injury (N=14, male=11, female=3)	*p*
**Height (m)**	1.73±0.1	1.74±0.1	*p*=0.86
**Body mass (kg)**	71.6±9.9	73.9±11.6	*p*=0.57
**Age (years)**	25±2.8	25±7.2	*p*=0.94

**Table 2:** Statistics for posturography measures obtained under different conditions of postural difficulty for the ACL injury group and the control group.

Test	Control (median)	ACL injury (median)	*p*	Effect size (Cohen’s D)
**Eyes open**				
Mediolateral displacement (cm)	0.30	0.11	0.89	0.50
Anteroposterior displacement (cm)	2.98	2.46	0.64	0.86
Velocity of displacement (cm/s)	3.40	3.92	0.20	1.24
Area of displacement (cm^2^)	7.24	6.81	0.23	0.30
**Eyes closed**				
Mediolateral displacement (cm)	3.07	0.04	0.04*	0
Anteroposterior displacement (cm)	0.42	1.29	0.19	0.10
Velocity of displacement (cm/s)	8.40	7.73	0.88	0
Area of displacement (cm^2^)	18.77	20.83	0.38	0
**Flexion/extension (squat)**				
Mediolateral displacement (cm)	0.44	0.26	0.01*	0.81
Anteroposterior displacement (cm)	1,27	0.73	0.63	0.89
Velocity of displacement (cm/s)	9.45	9.27	1.00	0.32
Area of displacement (cm^2^)	21.79	24.52	0.53	6.99
**Kick**				
Mediolateral displacement (cm)	0.11	0.67	0.45	1.85
Anteroposterior displacement (cm)	1.01	1.16	0.87	0.23
Velocity of displacement (cm/s)	10.31	11.20	0.76	1.33
Area of displacement (cm^2^)	32.54	33.58	0.66	0.31
